# Comparative Evaluation of Titanium Versus Zirconia Implants for Their Clinical Outcomes: An Overview of Reviews

**DOI:** 10.7759/cureus.108219

**Published:** 2026-05-04

**Authors:** Uttam Shetty, Arushi Sharma, Swapnali Mhatre, Reema Srichand, Mridula Joshi, Mosam Trivedi

**Affiliations:** 1 Prosthodontics, Bharati Vidyapeeth Dental College and Hospital, Navi Mumbai, IND; 2 Prosthodontics &amp; Crown and Bridge and Implantology, Bharati Vidyapeeth Dental College and Hospital, Navi Mumbai, IND

**Keywords:** clinical implant outcomes, dental implants, implant biocompatibility, implant osseointegration, titanium implants, zirconia implants

## Abstract

The use of dental implants has revolutionized dentistry, with titanium and zirconia being the most commonly utilized materials. This article presents a comparative evaluation of titanium and zirconia implants, focusing on their mechanical, biological, and clinical properties. Although the success of dental implant procedures depends on multiple variables, there is still insufficient literature documenting a comprehensive comparative evaluation of titanium and zirconia implants across their different properties. Therefore, this umbrella review aims to compare and evaluate various properties of titanium and zirconia implants.

The intent of this article was to conduct an overview of systematic reviews (SRs) to provide a present review of different properties of titanium and zirconium implants; to analyze survival rate (SR), marginal bone loss (MBL), and/or probing depth (PD), bleeding on probing (BoP), pink esthetic score, and plaque index (PI); and to identify which has better clinical results among the titanium and zirconia implants.

PubMed, Google Scholar, and Embase were used as literature databases. SRs, with or without meta-analysis, were included. A comprehensive search, both manual and electronic, was performed to identify SRs published between 2014 and March 2023 that aligned with the aims of this overview. The methodological quality of the selected reviews was evaluated using the A Measurement Tool to Assess Systematic Reviews (AMSTAR-2) tool. Data were primarily presented descriptively and supported by detailed tables summarizing characteristics and findings at both the review and trial levels.

The findings indicate that titanium implants demonstrate higher SRs (92.6%-100%) compared to zirconia implants (87.5%-91.25%), with zirconia associated with a greater risk of failure and lower overall success rates. MBL was comparable between the two groups (Ti: −1.17 to −0.125 mm; Zr: −1.38 to −0.25 mm), as were PDs (Ti: 1.6-3.05 mm; Zr: 2.21-2.6 mm), PI, and BoP (Zr: 16.43%; Ti: 10%-20%). Zirconia implants exhibited superior esthetic outcomes and greater early bone apposition at two weeks, whereas titanium implants showed a more favorable bone response at four weeks. Overall, titanium implants remain the gold standard for long-term predictability, while zirconia implants serve as a viable alternative in esthetically demanding cases.

## Introduction and background

Dental implants can restore chewing, speech, and esthetic functions effectively; this has established them as a reliable method for restoring lost dentition. Dental implants are now a necessary component of the dental armamentarium [1]. Considering elements like patient selection, thorough treatment planning, clinician expertise, and patient-focused outcomes, titanium (Ti) implants have demonstrated consistent effectiveness across multiple decades, solidifying their role as a dependable treatment option [2,3].

Clinical evaluations of implants have frequently examined their survival, failure, and success rates as well as their esthetics, peri-implant soft-tissue alterations, marginal bone level changes, and biological and mechanical complications [4]. Ti shows mechanical properties, superior biocompatibility, and promising long-term survival rates; therefore, it has become the principal material used in dental implantology. Achieving optimal esthetics is one of the major clinical concerns; the grayish hue of Ti implants can lead to discoloration of the peri-implant mucosa in individuals with a thin gingival phenotype [5]. Bone loss and stress shielding may be due to mechanical and elastic modulus differences between bone and the Ti implant. It may also lead to allergic reactions; hypersensitivity may occur due to corrosion of the implant surface, may lead to plaque formation and accumulation, and may also lead to peri-implantitis. To overcome these problems, zirconium (Zr) dioxide implants were introduced; the color of this was closer to the natural tooth, and this was light transmissive [5,6].

Zr has garnered considerable interest in the advancement of dental implant materials, owing to its exceptional biocompatibility, favorable esthetic qualities, robust corrosion resistance, commendable mechanical strength, and lack of documented hypersensitivity reactions [7]. In addition, Zr implants were developed to address the esthetic and biological limitations sometimes associated with Ti-based systems. Zr implants may demonstrate a comparatively slower onset of osseointegration than their Ti counterparts [8,9]. This study set out to systematically compare the mechanical strength and esthetic appeal of Ti versus Zr implants.

Objective

This overview of reviews aimed to determine the comparison of different properties of Ti and Zr implants. The primary outcomes of interest are survival rate, bleeding on probing (BoP), marginal bone loss (MBL), pocket depth (PD), and osseointegration. The secondary outcomes of interest are plaque index (PI), pink esthetic scale (PES), and reason for failure.

This umbrella review provides a high-level synthesis of various interventions targeting a particular condition or associated risk factor. It conserves valuable research resources by leveraging existing systematic reviews, eliminating the need to conduct systematic searches from the ground up.

## Review

Materials and methods

The development of this overview protocol followed the Preferred Reporting Items for Systematic Reviews and Meta-Analyses (PRISMA) guidelines [10].

PICOS Question and Eligibility Criteria

Studies were selected for this review based on the following inclusion criteria, established according to the PICOS framework (Table 1) [11].

**Table 1:** PICO for synthesis [11]

PICOS element	Description
Participants (P)	Patients with one or more missing teeth who have undergone partial or complete oral rehabilitation using at least two dental implants-titanium, zirconia (Zr)/titanium hybrid, or Zr implants
Intervention (I)	Veneered or monolithic Zr dental implants
Comparison (C)	Traditional titanium dental implants
Outcomes (O)	Primary outcomes: implant survival rate, bleeding on probing (BoP), marginal bone loss (MBL), probing pocket depth (PPD), and extent of osseointegration. Secondary outcomes: plaque index (PI), pink esthetic score (PES), and contributing factors to implant failure
Study period/timing (S)	Studies published from 2014 to March 2023

Research Question

In adult patients undergoing dental implant placement (P), how do Zr implants (I) compare to traditional Ti implants (C) in terms of long-term osseointegration success, peri-implant tissue response, esthetic outcomes, and mechanical durability (O), as reported in systematic reviews and meta-analyses (S)?

Inclusion and Exclusion Criteria

The criteria for the study inclusion were as follows: systematic reviews with or without meta-analysis evaluating the outcomes of Zr and Ti implants. The exclusion criteria were the systematic review of in vitro studies and the systematic review of animal studies. Other forms of studies such as randomized controlled studies, laboratory studies, case reports, uncontrolled trials, duplicate reviews, and editorials were excluded.

A comprehensive and systematic literature search was conducted to identify all relevant studies evaluating Zr and Ti dental implants. The search was performed across the following electronic databases: PubMed/MEDLINE, Embase, Cochrane Library, and Google Scholar (for gray literature). The search strategy combined controlled vocabulary (MeSH terms) and free-text keywords related to dental implants, Zr, Ti, and clinical outcomes.

The following keywords were used: dental implants, oral implants, implant-supported prosthesis, zirconia implants, zirconium implants, ceramic implants, Zr implants, titanium implants, Ti implants, conventional implants, implant survival, osseointegration, marginal bone loss, bleeding on probing, probing depth, peri-implant tissue, plaque index, esthetic outcomes, pink esthetic score (PES), and implant failure. For example, the keywords used in search were ("dental implants"[MeSH Terms] OR "oral implants" OR "implant-supported prosthesis") AND ("zirconia implants" OR "zirconium implants" OR "ceramic implants") AND ("titanium implants" OR "Ti implants") AND ("implant survival" OR "osseointegration" OR "marginal bone loss" OR "bleeding on probing" OR "probing depth" OR "peri-implant tissue" OR "esthetic outcomes"). Filters were applied to include only systematic reviews and systematic reviews and meta-analyses (SRMA) published in the English language.

**Figure 1:**
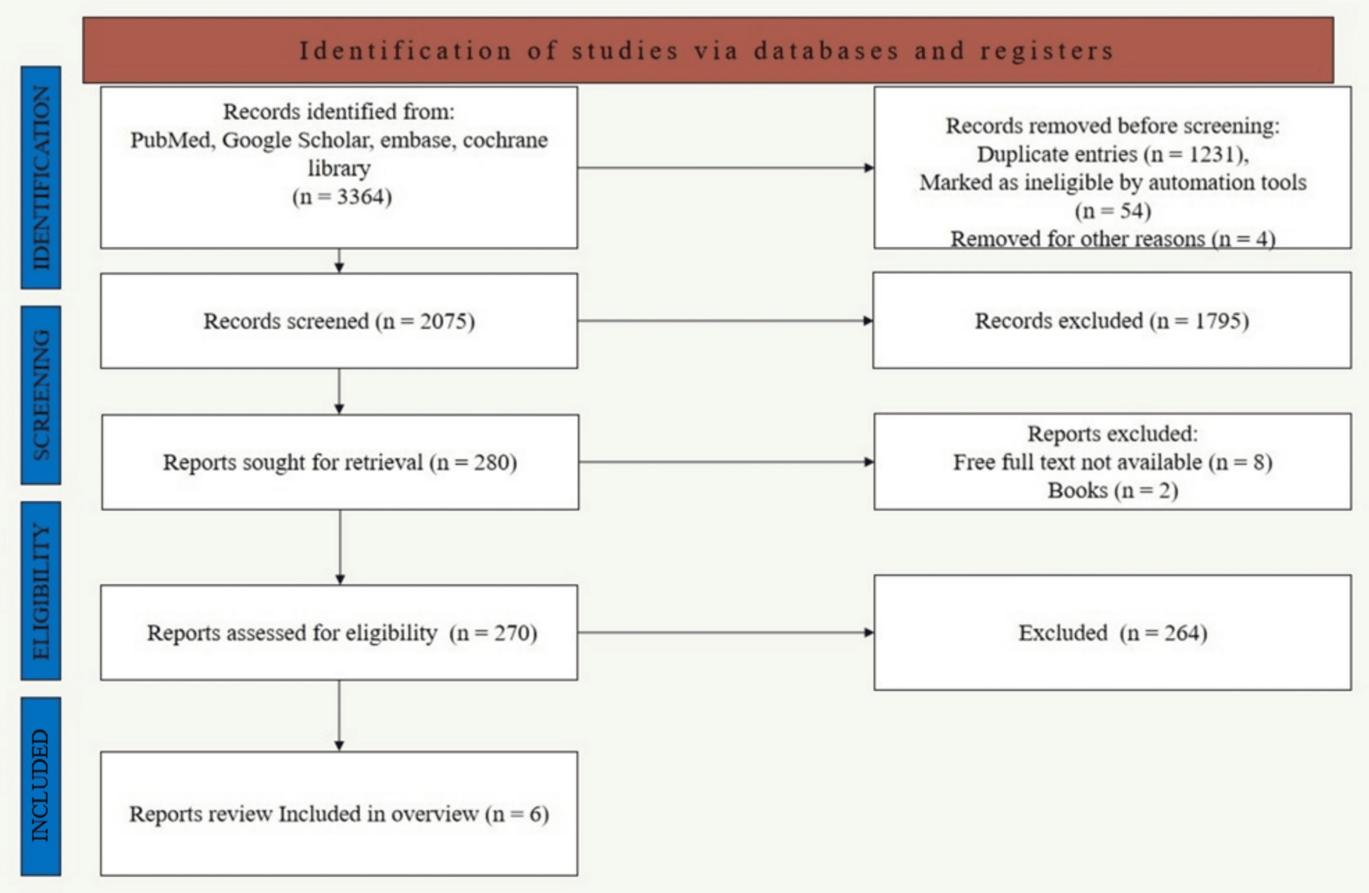
Flowchart showing the selection process of the systematic literature overview Image Credits: Dr. Arushi Sharma. Created using Microsoft 365 (Microsoft Corp., Redmond, WA, USA)

The titles and abstracts of all articles identified through the electronic database search were independently screened. The full texts of the potentially relevant articles were subsequently retrieved and assessed in detail, as shown in Figure 1. Disagreements were resolved through discussion. Reasons for excluding full-text articles were documented. Prior to inclusion, selected studies were meticulously cross-checked using details such as study center, ethical approval identifiers, and full author rosters to ensure that duplicate publications from the same trial were not mistakenly included.

Study Selection

Systematic reviews that assessed the differences between Ti and Zr implants with a minimum of six months of follow-up were analyzed. The combined manual and electronic search yielded 3,364 articles. Before screening, 1,231 duplicate records were removed using EndNote X9 (Clarivate, London, UK), 54 records were marked as ineligible by automated tools, and four records were removed for other reasons, including abstracts only and incomplete data. Following the preliminary screening of titles and abstracts, 1,795 records were excluded from further consideration. A total of 16 reports were assessed for eligibility out of 280 articles. Two authors (US and AJ) independently assessed each selected paper for inclusion in this comprehensive evaluation. Any disagreements were resolved through discussion with a third investigator (SM) and by consensus. The remaining six articles were reviewed in full and subsequently included in the study.

The methodological quality of the included systematic reviews was evaluated using the A Measurement Tool to Assess Systematic Reviews (AMSTAR-2) checklist [12]. Among the six reviews included in the umbrella review, four demonstrated high methodological quality, one showed moderate quality, and one review was rated as low quality. Earlier reviews showed methodological limitations such as absence of protocol registration and lack of duplicate study selection, whereas more recent reviews demonstrated stronger methodological rigor including comprehensive literature searches, risk-of-bias assessment, and appropriate meta-analysis methods (Table 2).

**Table 2:** Assessment of the methodological quality of the systematic reviews AMSTAR-2: A Measurement Tool to Assess Systematic Reviews [12]

AMSTAR-2 item	Duan et al., 2023 [13]	Fernandes et al., 2022 [14]	Elnayef et al., 2017 [15]	Padhye et al., 2023 [16]	Sales et al., 2023 [17]	Sivaraman et al., 2018 [18]
1. PICO clearly defined	Yes	Yes	Yes	Yes	Yes	Yes
2. Protocol registered before review	Yes	Yes	No	Yes	Yes	No
3. Study designs justified	Yes	Yes	Partial yes	Yes	Yes	Partial yes
4. Comprehensive literature search	Yes	Yes	Yes	Yes	Yes	Yes
5. Study selection in duplicate	Yes	Yes	Yes	Yes	Yes	No
6. Data extraction in duplicate	Yes	Yes	Yes	Yes	Yes	No
7. List of excluded studies	Yes	Partial yes	Partial yes	Yes	Partial yes	No
8. Description of included studies	Yes	Yes	Yes	Yes	Yes	Yes
9. Risk-of-bias assessment	Yes	Yes	Yes	Yes	Yes	Partial yes
10. Funding sources for included studies	Partial yes	Partial yes	No	Partial yes	Partial yes	No
11. Appropriate meta-analysis methods	Yes	Yes	Yes	Yes	Yes	N/A
12. Impact of bias on results	Yes	Yes	Partial yes	Yes	Yes	No
13. Heterogeneity discussion	Yes	Yes	Yes	Yes	Yes	Yes
14. Publication bias assessment	Yes	Yes	Yes	Yes	Yes	No
15. Conflict of interest reported	Yes	Yes	Yes	Yes	Yes	Yes
16. Review methods appropriate	Yes	Yes	Yes	Yes	Yes	Partial yes
Overall quality	High	High	Moderate	High	High	Low

Study Characteristics

Table 3 outlines the fundamental attributes of the studies included, which span publications from 2014 to 2023 and report an average follow-up duration of 11 months.

**Table 3:** Characteristics of the included studies MBL: marginal bone loss; PPD: probing pocket depth; PI: plaque index; BoP: bleeding on probing; PES: pink esthetic score

Sr. No.	Study	Location	Follow-up (months)	Outcomes
1)	Duan et al., 2023 [13]	China	12	Survival rate, success rate, MBL, PPD, PI, BoP, PES
2)	Fernandes et al., 2022 [14]	USA	6	Survival rate, MBL, PPD, PI, BoP, PES
3)	Elnayef et al., 2017 [15]	USA	12	Survival rate, success rate, MBL
4)	Padhye et al., 2023 [16]	UK	12	Success rate, survival rate, PES
5)	Sales et al., 2023 [17]		12	Survival rate, MBL, BoP, PPD
6)	Sivaraman et al., 2018 [18]	USA	12	Survival rate, success rate, MBL

A comparative evaluation of various parameters between Ti and Zi implants is detailed in Table 4.

**Table 4:** Assessment of the differences between titanium (Ti) and zirconia (Zr) implants MBL: marginal bone loss; PPD: probing pocket depth; PI: plaque index; BoP: bleeding on probing; PES: pink esthetic scale; RCT: randomized controlled trial; RR: risk ratio; TZ: titanium-zirconium

Sr. No.	Study	Survival rate	Success rate	MBL	PPD	PI	BoP	PES	Osseointegration
1)	Duan et al. (2023)-included 4 RCTs (248 implants) with up to 5-year follow-up [13]	Ti > Zr 4/4 studies; Zr implants showed a lower survival rate (RR 0.91; CI 0.82-1.02; p = 0.10; I² = 0%), with survival rates of 92.77%, 98.58%, and 86.38% at 1-, 2-, and 5-year follow-up	Ti>Zr 4/4 studies Zr implants showed a significantly lower success rate than Ti (RR 0.87; CI 0.78-0.98; p = 0.03; I² = 0%), with success rates of 84.89%, 80.78%, and 66.38%. 1-, 2-, and 5-year follow-up	No significant difference between Ti and Zr implants	Ti = Zr 3/3 studies	Ti > Zr 1/3 studies	Ti > Zr 2/2 studies	No significant difference between Ti and Zr implants	-
2)	Fernandes et al. (2022)-included 7 studies. A total of 646 implants (169 Zr, 194 TZ, and 283 Ti) with a follow-up of 12-80 months [14]	Ti > Zr 5/7 studies; survival rate ranged from 92.6%-100% (Ti), 95.8%-100% (TZ), and 87.5%-91.25% (Zr)	-	Ti = Zr 7/7 studies; MBL ranged from −1.17 to −0.125 mm (Ti), −0.6 to −0.32 mm (TZ), and −1.38 to −0.25 mm (Zr)	Ti = Zr 3/3 studies; PD ranged from 1.6-3.05 mm (Ti), 3.12 mm (TZ), and 2.21-2.6 mm (Zr)	Ti = Zr 5/7 studies; low incidence of mucositis and peri-implantitis with Zr implants	Ti = Zr 4/4 studies; BoP was 16.43% (Zr) and 10%-20% (Ti)	-	-
3)	Elnayef et al. (2017)-included 21 articles (1,948 implants) with up to 40-month follow-up [15]	Ti > Zr 3/3 studies; Zr implants had 89% higher failure than Ti	Ti = Zr 2/2 studies	Ti	-	-	-	-	-
4)	Padhye et al. (2023)-included 4 studies (100 Zr, 99 Ti implants) with 12-month follow-up [16]	Ti = Zr 2/2 studies	Ti > Zr 2/2 studies; Zr success rates ranged from 57.5% to 93.3%	-	-	-	-	Ti > Zr 2/2 studies	-
5)	Sales et al. (2023)-included 3 studies (71 patients; 192 implants-87 Ti, 105 Zr) with 12-80 months follow-up [17]	Ti > Zr 3/3 studies; effectiveness was 87.4% (Ti) and 78.1% (Zr)	-	-	-	-	-	-	-
6)	Sivaraman et al. (2018)-included 47 studies [18]	Ti > Zr 13/13 studies	Ti = Zr 8/13	Ti = Zr 7/10	-	-	-	-	Ti = Zr 6/6 studies; at 2 weeks, Zr showed higher bone apposition (54%-55% vs. 42%-52% Ti), while at 4 weeks, Ti was higher (68%-91% vs. 62%-80% Zr)

Data Synthesis

Survival rate: Six articles included in this review compared the survival rate of Ti and Zr implants, and in five articles, it was found that the survival rate ranged from 87.5% to 93.3% in Zr, while Ti implants showed higher survival rates between 92.6% and 100% [13-15,17,18]. In one of the articles, it was seen that the survival rate of both implants was equal [16]. These values were primarily assessed through longitudinal clinical follow-up of randomized controlled trials (RCTs) and controlled clinical trials (CCTs), with survival defined as the presence of the implant in situ over the study period. Meta-analyses (e.g., Duan et al. [13] and Elnayef et al. [15]) used risk ratios (RRs) and odds ratios (ORs) to quantify differences, while others like Fernandes et al. [14] and Padhye et al. [16] reported descriptive survival percentages across follow-up periods ranging from 12 to 80 months. Although Zr implants demonstrated acceptable short-term survival, Ti implants remain the more reliable option for long-term outcomes.

Success rate: Only four authors compared the success rate of both implants, and it was found that the success rate for Ti implants was greater according to two studies (Duan et al. [13] and Padhye et al. [16]), while the studies by Elnayef et al. [15] and Sivaraman et al. [18] found that the success rate of Ti and Zr implants was equal. The reported success rates for Zr implants ranged from 57.5% to 93.3%, while Ti implants ranged from 57.1% to 100%. These values were derived from RCTs and CCTs, with success defined by criteria such as absence of pain, infection, mobility, radiolucency, and peri-implant bone loss. Meta-analyses (e.g., Duan et al. [13] and Elnayef et al. [15]) used RRs and ORs to compare outcomes, while others (e.g., Fernandes et al. [14] and Padhye et al. [16]) reported descriptive success percentages over follow-up periods ranging from 12 to 80 months. Elnayef et al. reported that Zr implants had an approximately 89% higher failure risk compared to Ti implants [15]. Although Zr implants demonstrated acceptable clinical success, Ti implants consistently showed broader and more stable success, reinforcing their reliability in diverse scenarios.

Marginal bone loss: MBL was compared in four studies, and according to two authors, it was found that MBL was greater with Zr implants (Duan et al. [13] and Elnayef et al. [15]). This was because factors like less surface roughness, one-piece limitations, biomechanical rigidity, and technique sensitivity can lead to higher MBL. According to two studies by Fernandes et al. [14] and Sivaraman et al. [18], MBL was equal for both implants. An initial MBL of up to 1.5 mm within the first year is considered within acceptable limits, with a permissible annual reduction not exceeding 0.2 mm in the years that follow. Exceeding this range may indicate peri-implant disease, biomechanical overload, or poor implant integration.

Probing pocket depth: Only two of the included studies compared the probing PD (PPD), and according to Duan et al. [13], PPD was greater with Ti implants, but in a study by Fernandes et al. [14], PPD was seen to be similar in both Ti and Zr implants.

Plaque index: Two authors compared the PI, and Duan et al. [13] favored Zr implants, as the PI was greater in patients with Ti implants, while no significant difference was found between the implants in a study by Fernandes et al. [14]. These values were measured using standardized periodontal indices and visual scoring systems during clinical follow-up in RCTs and comparative studies. Overall, both materials demonstrated comparable plaque control outcomes, with Zr showing a potential advantage due to its smoother surface and lower bacterial affinity.

Bleeding on probing: Duan et al. [13] observed higher BoP levels in Ti implants. These measurements were obtained using calibrated periodontal probes during routine clinical evaluations in RCTs and comparative studies. In contrast, Fernandes et al. [14] found no statistically significant difference in BoP between Ti and Zr implants. Higher BoP observed around Ti implants may be attributed to increased plaque accumulation and bacterial adhesion on their relatively rough surfaces, along with a comparatively less favorable soft-tissue response, whereas Zr implants exhibit reduced biofilm formation and enhanced mucosal integration, resulting in lower peri-implant inflammation.

Pink esthetic score: Assessment was conducted on the position of the gingival margin, the contour of the labial surface, and the color and texture of the tissue surrounding the implant. Among the six studies included in this review, only two authors compared the PES. Duan et al. [13] reported higher PES values with Zr implants, whereas Padhye et al. [16] observed greater PES with Ti implants. These scores were assessed using standardized clinical photographs and PES criteria, which evaluate soft-tissue parameters like papilla fill, contour, and mucosal color.

Osseointegration: One study compared the rate of osseointegration between Ti and Zr implants, and according to Sivaraman et al. [18], osseointegration was comparable for both Ti and Zr implants. They reviewed some in vitro studies that reported bone-to-implant contact (BIC%), which is a direct histological measure of osseointegration. In human clinical research, indirect measures like implant survival, MBL, and the condition of peri-implant tissues were used as key indicators.

Discussion

The mechanical characteristics of dental biomaterials are pivotal in influencing their functional outcomes and durability in restorative procedures. A thorough assessment of both mechanical and esthetic features-especially in Ti and Zr-offers critical insights into their clinical performance and appropriateness across diverse treatment scenarios [19].

Dental implants have revolutionized restorative dentistry by providing dependable options for tooth replacement. Among the materials commonly employed, Ti and Zr stand out due to their distinct characteristics, which significantly impact clinical performance. The choice of material is essential in prosthodontics, as it can affect the long-term success of the implant, esthetic outcomes, patient comfort, and cost. In this article, we have compared Ti and Zr prosthodontic implants, focusing on their biocompatibility, mechanical properties, esthetics, osseointegration, clinical performance, and overall application in modern prosthodontics.

Ti has long been favored in dental implantology owing to its superior biocompatibility. Its clinical success is largely attributed to the spontaneous formation of a Ti dioxide (TiO₂) layer on the surface, which acts as a barrier against corrosion and facilitates osseointegration-the direct bonding of bone to the implant. This oxide layer also plays a crucial role in reducing the likelihood of adverse tissue responses [20]. Ti hypersensitivity is considered rare compared to other metals such as nickel, and when it does occur, it is typically a Type IV delayed (cell-mediated) immune reaction. The low incidence of sensitivity is largely attributed to the presence of a stable TiO₂ layer on the implant surface, which is chemically inert and acts as a protective barrier, preventing the release of ions into surrounding tissues. This limits direct interaction with the immune system and reduces the likelihood of sensitization. However, certain host-related factors, including a history of metal allergies or altered immune responses, may increase individual susceptibility.

Zr, specifically yttria-stabilized Zr (YSZ), has emerged as a strong alternative to Ti in prosthodontics. Zr is also biocompatible, showing excellent compatibility with human tissue and bone. Its smooth surface reduces the risk of bacterial adhesion, which is a critical factor in the prevention of peri-implant infections. Zr does not release harmful ions into the body, and it is non-reactive, which contributes to its overall biocompatibility [21]. For patients prioritizing esthetics, particularly in the upper front region, Zr implants offer a significant advantage over Ti.

The findings of this study clearly show that different materials possess distinct mechanical properties, affecting their appropriateness for particular clinical uses. Ti exhibits superior tensile strength, reflecting its mechanical resilience when compared to Zr and other ceramic-based materials. This is consistent with the study by Bojko et al., who assessed the static behavior of Ti alloys via axial tensile testing and documented high ultimate tensile strength values [22].

Zr is characterized by the highest elastic modulus among commonly used implant materials, signifying its pronounced resistance to structural deformation. Supporting this, Ozdogan and Yesil Duymus [23] found that grinding markedly improved the hardness of Zr ceramics, emphasizing their durability against indentation and wear.

In contrast, Ti demonstrates superior fatigue resistance, offering enhanced long-term mechanical stability compared to Zr and ceramic. Thoma et al. [24] reported that following six months of functional loading, the peri-implant soft-tissue contours around one-piece Ti implants remained comparable to those surrounding Zr implants.

Zr exhibits superior hardness, reflecting its strong resistance to wear and indentation. This was substantiated by Zidan et al. [25], who observed a marked increase in surface hardness when polymethyl methacrylate (PMMA) was reinforced with Zr nanoparticles, outperforming a nanocomposite formulation containing silanized Zr. In a follow-up investigation, the same authors demonstrated Zr’s capacity to enhance mechanical properties, revealing that its incorporation into conventional denture base resins significantly elevated flexural strength, impact resistance, and surface hardness [26].

When the survival rate is compared, it was seen that Ti implants have a better survival rate than the Zr implants [13,14,18]. Therefore, the study supports the use of Ti implants. In the overall analysis of MBL, the findings favored the use of Ti implants. The PPD, PI, and BoP were greater with Ti implants. Despite observed differences, their clinical relevance remains uncertain due to non-equivalent baseline parameters and minimal variance. Ti implants demonstrated superior osseointegration, reinforcing the preference for Ti as the material of choice in prosthetic applications. Its favorable attributes contribute to enhanced survival and success rates, improved PES, and reliable osseointegration outcomes.

Limitations

This umbrella review is limited by its qualitative narrative synthesis without quantitative pooling and the small number of included systematic reviews due to availability constraints. Additionally, overlap of primary studies, heterogeneity in methodologies and outcomes, and variable review quality may affect comparability and the strength of conclusions; future studies should include broader literature access, apply quantitative synthesis where appropriate, standardize outcomes, and carefully manage study overlap to strengthen evidence reliability.

## Conclusions

Both Ti and Zr implants have unique advantages and limitations depending on the specific clinical situation. Ti remains the gold standard for its well-established history, mechanical properties, and clinical success rates. It is particularly suited for load-bearing applications and has a long track record of osseointegration.

Zr implants offer significant esthetic benefits, especially for anterior dental applications, due to their tooth-like appearance. However, their mechanical properties and long-term performance still require more extensive research to match the success of Ti implants in some cases. In practice, the choice between Ti and Zr implants should be based on the patient’s individual needs, the location of the implant, esthetic concerns, and cost considerations.
